# Small Bowel Obstruction Due to Concurrent Petersen’s and Pouch of Douglas Hernias in a Patient With a Complex Surgical History: A Rare Surgical Case

**DOI:** 10.7759/cureus.77219

**Published:** 2025-01-10

**Authors:** Abigayle Wyer, Mena Louis, Nathaniel Grabill, Bradley Kuhn

**Affiliations:** 1 Surgery, Northeast Georgia Medical Center Gainesville, Gainesville, USA; 2 General Surgery, Northeast Georgia Medical Center Gainesville, Gainesville, USA; 3 Trauma and Acute Care Surgery, Northeast Georgia Medical Center Gainesville, Gainesville, USA

**Keywords:** abdominal hernia, gastric bypass, petersen's hernia, pouch of douglas, small bowel obstruction

## Abstract

Internal hernias are a rare but significant cause of small bowel obstruction, particularly in patients with a history of abdominal surgery such as Roux-en-Y gastric bypass (RYGB). Although Petersen’s hernia is the most commonly encountered internal hernia in these patients, herniation into the Pouch of Douglas is an exceedingly rare occurrence. This report describes the case of a 77-year-old female with a complex surgical history, including RYGB and hysterectomy, who presented with several months of postprandial abdominal pain, nausea, and vomiting. A computed tomography (CT) scan initially suggested mild bowel distention without clear evidence of obstruction. However, due to the inability to tolerate oral intake, a follow-up CT scan was performed and revealed dilated loops of the small bowel, prompting surgical intervention. Intraoperatively, two internal hernias were identified: one at Petersen’s defect and another in the Pouch of Douglas, the latter being the cause of the obstruction. Both hernias were reduced, and the peritoneal defect in the Pouch of Douglas was closed using sutures. The patient recovered without complications and was discharged to rehabilitation. This case demonstrates the importance of considering internal hernias, including rare types, in post-surgical patients presenting with nonspecific symptoms of bowel obstruction. Early diagnosis and timely surgical management are crucial to prevent complications such as bowel ischemia and to ensure optimal outcomes. Closing peritoneal defects during hernia repairs is essential to minimize the risk of recurrence. This case contributes to the limited literature on internal hernias involving the Pouch of Douglas and emphasizes the need for thorough diagnostic evaluation in complex clinical scenarios.

## Introduction

Internal hernias are a relatively rare but important cause of small bowel obstruction (SBO), with an incidence ranging from 0.2% to 5.8% of all intestinal obstructions [1]. An internal hernia can occur when a portion of the intestine protrudes through a mesenteric or peritoneal defect within the abdominal cavity, leading to bowel strangulation, ischemia, or even necrosis if left untreated. These hernias are particularly difficult to diagnose due to their nonspecific clinical presentation, often mimicking other causes of bowel obstruction such as adhesions or external hernias [2]. Computed tomography (CT) has become an indispensable tool in the early identification of internal hernias, though a definitive diagnosis is often made intraoperatively [3]. 

One of the most recognized sites for internal herniation in post-surgical patients is Petersen’s space, especially in those who have undergone Roux-en-Y gastric bypass (RYGB) surgery [4]. RYGB is a weight loss procedure where the bowels are re-routed to bypass part of the stomach and result in a mesenteric defect that is prone to internal hernias. Petersen’s hernia occurs when the small bowel herniates through the mesenteric defect created during this procedure, which can occur years after surgery [5]. RYGB is a well-established risk factor for internal hernias, with studies indicating that 1% to 5% of patients may develop this complication postoperatively [6]. The clinical presentation of Petersen’s hernia is often vague, with patients reporting intermittent abdominal pain, nausea, and vomiting, sometimes only after meals [7]. This nonspecific presentation can delay diagnosis, further increasing the risk of complications such as bowel strangulation and ischemia [8]. 

Another, much rarer, location for internal herniation is the Pouch of Douglas, also known as the rectouterine pouch in females, which lies between the rectum and the uterus [9]. Herniation into this space is exceedingly uncommon, with fewer than a dozen cases reported in the literature [10]. The Pouch of Douglas is typically involved in cases of pelvic hernias, and defects in this region can arise either congenitally or following surgical procedures such as hysterectomy [11]. The rarity of internal hernias in this location further complicates their diagnosis as they are often overlooked in differential diagnoses of SBO [12]. A high index of suspicion is required to identify these hernias, especially in post-surgical patients with nonspecific symptoms of bowel obstruction. 

The management of internal hernias typically involves surgical intervention, with laparoscopy often being the first-line approach [13]. However, due to the potential complexity and difficulty in reducing hernias laparoscopically, conversion to open surgery is not uncommon [14]. Surgical reduction of the herniated bowel and closure of the peritoneal defect are critical to prevent recurrence and avoid severe complications such as bowel ischemia or necrosis [15]. The decision to close the defect is supported by the literature, which suggests that closure significantly reduces the likelihood of recurrent herniation [16]. Furthermore, timely diagnosis and intervention are paramount as delayed treatment increases morbidity and mortality. 

## Case presentation

A 77-year-old female with a significant medical history, including RYGB, hypertension, atrial fibrillation, hypothyroidism, chronic kidney disease (CKD), diabetes mellitus, hyperlipidemia, and a pacemaker for sick sinus syndrome, presented to the emergency department (ED) with complaints of abdominal pain, nausea, and vomiting. She admitted to having small bowel movements within a couple of days prior to her presentation to the ED. The symptoms had persisted for several months, with the pain typically occurring 30 minutes after eating. Additionally, she reported rectal pain, which she associated with an enema administered a few days prior to her presentation. On physical examination, her abdomen was soft, non-tender, and non-distended, without palpable masses or signs of peritonitis. A perirectal exam revealed hemorrhoids but no erythema or tenderness. 

Laboratory investigations were performed upon admission, revealing a white blood cell count (WBC) of 11.5 × 10^9^/L, mild anemia with hemoglobin (Hgb) of 9.7 g/dL, hypokalemia with potassium (K) of 3.2 mmol/L, and a creatinine (Cr) level of 1.27 mg/dL, consistent with her underlying CKD. 

Initial imaging

**Figure 1:**
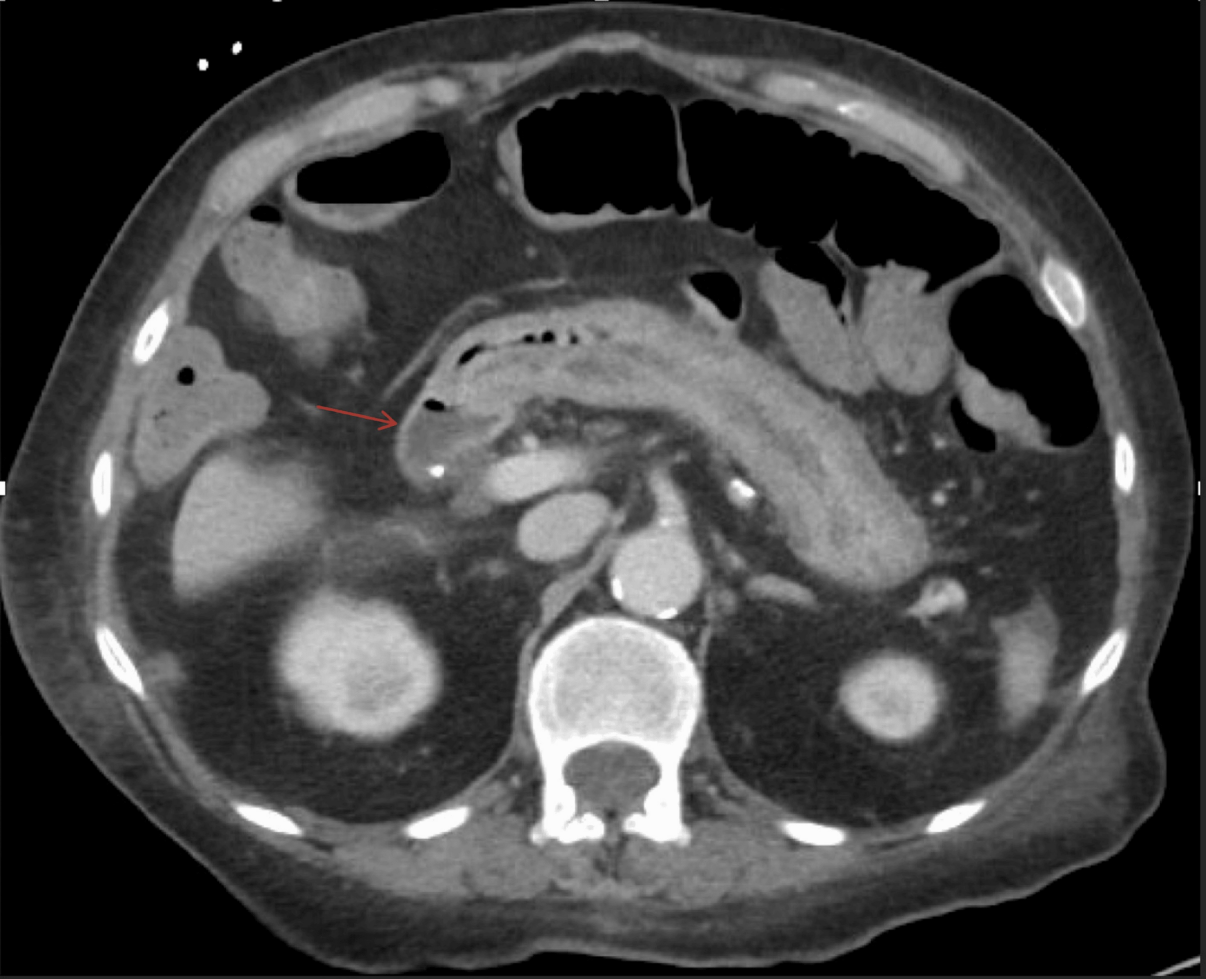
CT abdomen and pelvis with IV contrast (axial view) demonstrates a history of prior gastric bypass surgery with mild distention of small bowel loops on the right side (red arrow). CT: computed tomography; IV: intravenous

A CT scan of the abdomen and pelvis obtained on the day of admission demonstrated a history of prior gastric bypass surgery with mild distention of small bowel loops on the right side (Figure 1). The scan also revealed circumferential rectal wall thickening and fat stranding, possibly reflecting proctitis. In addition, a small fluid collection near the perineum, consistent with a perirectal abscess, was noted. No complete mechanical bowel obstruction was identified. 

**Figure 2:**
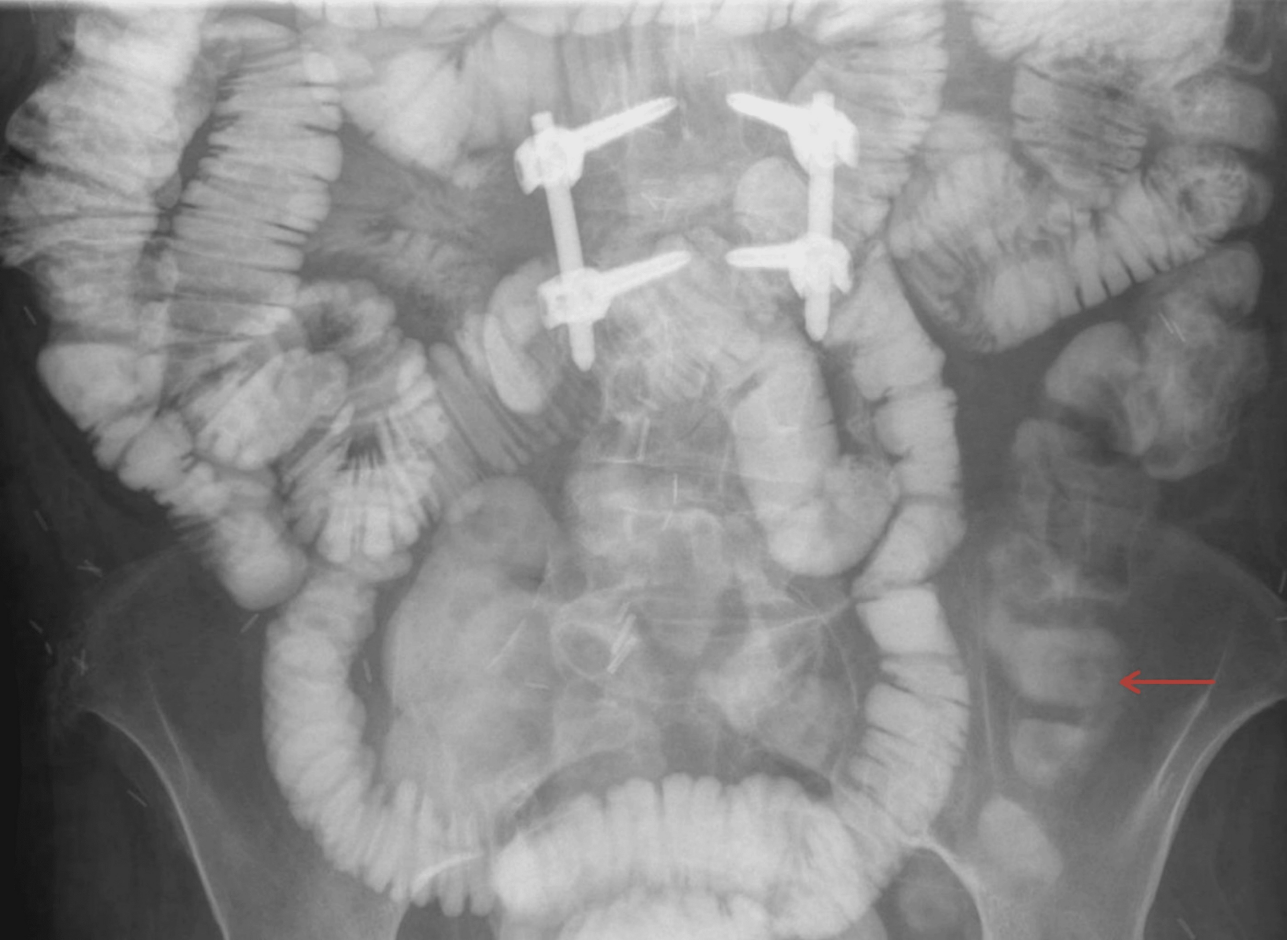
SBFT shows mild bowel distention consistent with ileus (red arrow), without definitive evidence of obstruction. SBFT: small bowel follow-through

A small bowel follow-through (SBFT) performed later showed mild bowel distention consistent with ileus, without definitive evidence of obstruction (Figure 2). Despite these findings, the patient’s abdominal pain and symptoms persisted. 

Clinical course

**Figure 3:**
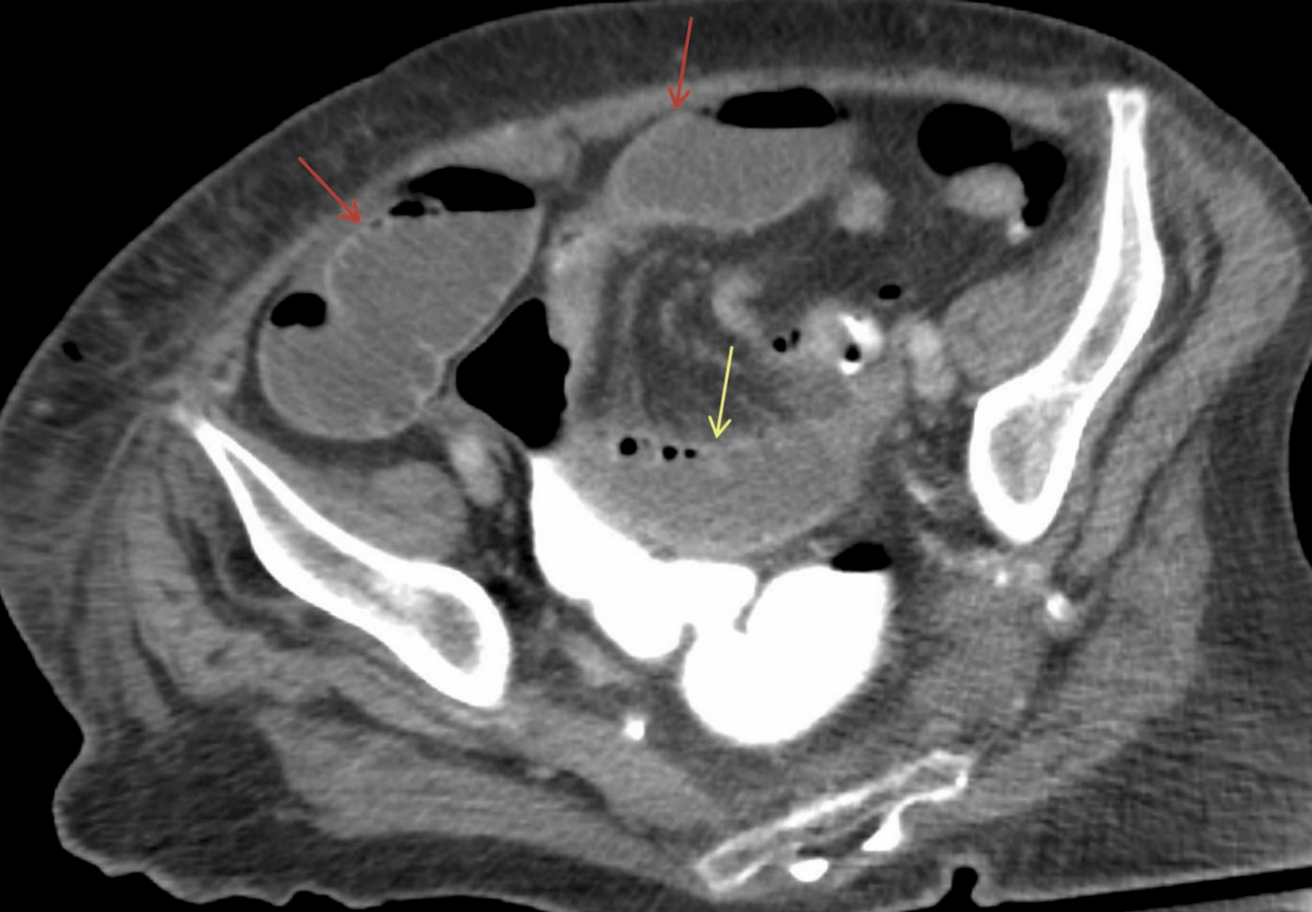
CT abdomen and pelvis with IV contrast (axial view) demonstrates dilated loops of small bowel along the anterior abdominal wall (red arrows) extending into the pelvis (yellow arrow), without evidence of any definite free air. CT: computed tomography; IV: intravenous

Over the course of the next three days, the patient’s symptoms worsened, prompting a repeat CT scan. This scan demonstrated dilated loops of small bowel, suggesting a partial bowel obstruction (Figure 3). With these findings, a surgical consultation was obtained and a diagnostic laparoscopy was scheduled. 

Surgical findings

A diagnostic laparoscopy was performed but was quickly converted to an open procedure due to the complexity of reducing the internal hernias. Intraoperatively, two internal hernias were identified. The first hernia involved Petersen’s defect, commonly associated with RYGB, although this was not the source of the obstruction. The second hernia was located in the Pouch of Douglas and was responsible for the bowel obstruction. 

The herniated small bowel was reduced from the Pouch of Douglas, and after inspection, the bowel was noted to be viable, with no signs of ischemia or necrosis. The defect in the Pouch of Douglas was closed using two 3-0 vicryl sutures in a purse-string fashion. The small bowel was further examined. The common limb, biliopancreatic limb, and Roux limb of the gastric bypass were intact, with no additional abnormalities. 

Postoperative course

Following the surgery, the patient was transferred to the post-anesthesia care unit (PACU) in stable condition. A nasogastric tube placed intraoperatively was left in place for decompression. Post-surgical labs showed a significant rise in the WBC to 25.6 × 10^9^/L, likely due to postoperative inflammation. Her Hgb improved to 10.6 g/dL, while K normalized to 4.6 mmol/L. Cr decreased to 1.10 mg/dL, indicating improved renal function. 

The patient tolerated the procedure well with gradual improvement in symptoms. She transitioned from nasogastric decompression to oral intake in the days following surgery and was discharged to a rehabilitation facility for continued recovery. 

Follow-up

At the time of discharge, the patient was tolerating a soft diet and reported no recurrence of her abdominal pain, nausea, or vomiting. She was scheduled for follow-up appointments with both the surgical and gastroenterology teams to monitor her recovery and overall health. 

## Discussion

Internal hernias are a rare but significant cause of SBO, particularly in patients with a history of abdominal surgery [17]. The overall incidence of internal hernias ranges from 0.2% to 5.8% of all cases of intestinal obstruction, and the presentation can be nonspecific, often delaying diagnosis and increasing the risk of complications such as bowel strangulation, ischemia, and necrosis [18]. This case highlights the unique challenges associated with diagnosing and managing internal hernias in a patient with a complex surgical history, specifically RYGB [19]. 

One of the most common forms of internal hernia following gastric bypass is Petersen’s hernia, which occurs when the small bowel herniates through the mesenteric defect created during the bypass procedure [5]. The incidence of Petersen’s hernia in RYGB patients ranges from 1% to 5%, and it typically presents with intermittent, postprandial abdominal pain, nausea, and vomiting symptoms very similar to those experienced by the patient in this case [20]. However, despite being a well-documented complication, Petersen’s hernia was not the cause of obstruction in this patient, though it was identified intraoperatively. 

The more significant finding in this case was the internal herniation into the Pouch of Douglas, also known as the rectouterine pouch. This is an extremely rare site for herniation, with fewer than a dozen cases reported in the literature [10]. The Pouch of Douglas is the lowest point of the peritoneal cavity in women, located between the rectum and the uterus [11]. Herniation into this space can occur either congenitally or as an acquired condition, particularly following pelvic surgeries such as hysterectomy, which this patient had previously undergone [19]. The presence of a prior hysterectomy may have weakened the pelvic floor or peritoneal structures, predisposing the patient to this rare form of internal hernia [20]. 

The literature on herniation into the Pouch of Douglas is sparse, but the few cases that have been reported suggest that it is a challenging diagnosis to make preoperatively. A review of cases by Suwa et al. (2013) describes the difficulty in diagnosing internal hernias based on imaging alone as the findings are often nonspecific, such as dilated loops of bowel or fluid collections, which are common to many causes of SBO [9]. In this case, initial imaging showed mild bowel distention and circumferential rectal wall thickening, with no definitive signs of full mechanical obstruction. Only on repeat imaging did dilated loops of the small bowel become apparent, suggesting partial obstruction and prompting surgical intervention. 

Once the decision for surgery is made, the choice of surgical approach - laparoscopic versus open - is a critical consideration. Laparoscopy is often the first-line approach due to its minimally invasive nature and shorter recovery times. However, in complex cases such as this, where significant tension is required to reduce the hernia, conversion to an open procedure may be necessary to avoid injury to the small bowel. In this case, laparoscopy was initially attempted but the hernia was not safely reducible without significant tension, necessitating an open approach. 

The decision to close the peritoneal defect in the Pouch of Douglas was crucial in preventing the recurrence of the hernia. The literature supports closing peritoneal defects during hernia repairs, as failure to do so may lead to recurrent herniation and further complications [20]. In this case, the defect was closed using 3-0 vicryl sutures in a purse-string fashion, and the postoperative course was uncomplicated, with the patient being discharged to rehabilitation in stable condition. 

This case also demonstrates the importance of early surgical intervention in preventing bowel ischemia or necrosis. Although no signs of ischemia were noted during surgery, the patient's delayed presentation and the progression of her symptoms over several months placed her at increased risk. Studies show that delays in the diagnosis and treatment of internal hernias are associated with higher rates of bowel resection and mortality. In this case, prompt recognition of the need for surgery likely contributed to the favorable outcome. 

## Conclusions

Internal hernias present diagnostic and surgical complexities, particularly in patients with a history of RYGB. While Petersen’s hernia is a common complication in these patients, rarer herniation sites like the Pouch of Douglas should be considered in cases of SBO. Early surgical intervention, including the closure of peritoneal defects, is crucial to prevent recurrence and complications such as bowel ischemia. Careful evaluation of post-surgical patients is necessary, even when initial imaging is inconclusive. 
